# Role and potential therapeutic value of histone methyltransferases in drug resistance mechanisms in lung cancer

**DOI:** 10.3389/fonc.2024.1376916

**Published:** 2024-03-08

**Authors:** Linxiang Zhang, Xueying Zhang, Yan Shi, Yuhan Ni, Jiaojiao Fei, Zhixin Jin, Wenjuan Li, Xiaojing Wang, Nan Wu

**Affiliations:** ^1^ Key Laboratory of Clinical and Preclinical Research in Respiratory Disease, Molecular Diagnosis Center, The Department of Pulmonary Critical Care Medicine, First Affiliated Hospital of Bengbu Medical College, Bengbu, Anhui, China; ^2^ Joint Research Center for Regional Diseases of Institute of Health and Medicine (IHM), The First Affiliated Hospital of Bengbu Medical College, Bengbu, China

**Keywords:** lung cancer, histone methyltransferases, epigenetics, drug resistance, cancer therapy

## Abstract

Lung cancer, ranking second globally in both incidence and high mortality among common malignant tumors, presents a significant challenge with frequent occurrences of drug resistance despite the continuous emergence of novel therapeutic agents. This exacerbates disease progression, tumor recurrence, and ultimately leads to poor prognosis. Beyond acquired resistance due to genetic mutations, mounting evidence suggests a critical role of epigenetic mechanisms in this process. Numerous studies have indicated abnormal expression of Histone Methyltransferases (HMTs) in lung cancer, with the abnormal activation of certain HMTs closely linked to drug resistance. HMTs mediate drug tolerance in lung cancer through pathways involving alterations in cellular metabolism, upregulation of cancer stem cell-related genes, promotion of epithelial-mesenchymal transition, and enhanced migratory capabilities. The use of HMT inhibitors also opens new avenues for lung cancer treatment, and targeting HMTs may contribute to reversing drug resistance. This comprehensive review delves into the pivotal roles and molecular mechanisms of HMTs in drug resistance in lung cancer, offering a fresh perspective on therapeutic strategies. By thoroughly examining treatment approaches, it provides new insights into understanding drug resistance in lung cancer, supporting personalized treatment, fostering drug development, and propelling lung cancer therapy into novel territories.

## 1 Introduction

Lung cancer, a prevalent malignant tumor, maintains high incidence and mortality rates globally (1). Data from 2020 show approximately 2.2 million new lung cancer cases worldwide, accounting for 11.4% of all new malignant tumor cases, ranking second after breast cancer. Lung cancer-related deaths are estimated at 1.8 million, representing 22% of all cancer fatalities, thus becoming the primary cause of cancer-related deaths (2). Lung cancer encompasses various types, including non-small cell lung cancer (NSCLC), which constitutes 85%, and small cell lung cancer (SCLC) (3). These types exhibit distinct clinical manifestations and treatment strategies (4), adding complexity to lung cancer treatment and underscoring the necessity for an in-depth understanding of its pathological and molecular basis. Conventional lung cancer treatments, such as surgical resection, radiotherapy, and chemotherapy (5), have improved patient survival to some extent. However, advances in molecular biology and genetics have fostered the development of new treatment strategies, like targeted therapy and immunotherapy, offering more personalized and innovative options for lung cancer patients (6–8). Targeted therapy, based on molecular targets for precisely treating tumor cells (9), selectively inhibits specific molecules in tumor cells, effectively impeding their growth and spread (9). Key targets in NSCLC, including epidermal growth factor receptor (EGFR), anaplastic lymphoma kinase (ALK), ros proto-oncogene 1 (ROS1), have become central to targeted therapy (10). Immunotherapy, by activating the patient’s immune system to attack tumor cells, enhances the capability of T cells and other immune cells to target cancer cells (11, 12). Although these therapeutic approaches bring new hope to the treatment of lung cancer, they still face significant challenges, particularly with regards to the issue of drug resistance (13). The emergence of drug resistance poses a major obstacle to precision medicine (14–16),limiting the effectiveness of traditional chemotherapy, targeted therapy, and immunotherapy (17). Therefore, understanding and overcoming drug resistance remains a crucial challenge in current lung cancer research (18).

Recent years have seen a growing focus on the role of epigenetic regulation in lung cancer drug resistance (19). Epigenetic regulation alters gene expression levels without involving DNA sequence changes (20). In this complex network, HMTs have garnered widespread interest due to their pivotal role in epigenetics, chiefly in regulating histone methylation modifications, influencing gene expression, and cellular signaling pathways (21). HMTs are aberrantly activated in various cancers, with their role in lung cancer drug resistance becoming increasingly prominent.

This review aims to thoroughly delineate the critical role of HMTs in lung cancer drug resistance, exploring their molecular mechanisms related to resistance. By systematically reviewing relevant studies, we aim to provide new insights into the molecular mechanisms underlying lung cancer drug resistance. We will also delve into therapeutic strategies targeting HMTs, including existing and developmental drugs. We will evaluate the potential efficacy of these strategies in overcoming lung cancer drug resistance and look forward to future research directions. By deeply exploring the relationship between HMTs and lung cancer drug resistance, we hope to support personalized lung cancer treatment and new drug development, offering more effective treatment options for patients.

## 2 HMTs and its function

**Figure 1:**
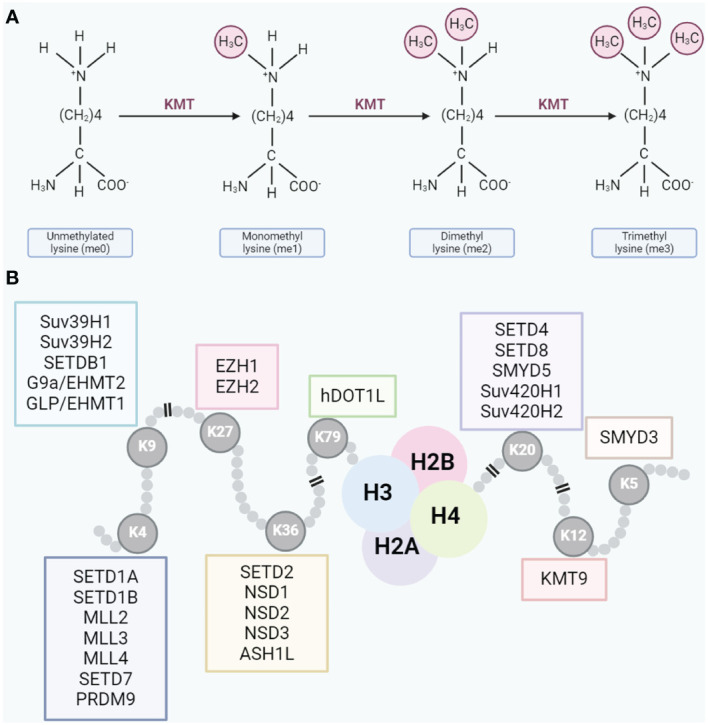
**(A)** Lysine residues can undergo mono-, di-, or trimethylation. **(B)** Typical methylation sites on lysine residues.

HMTs play a crucial role in cellular epigenetics, primarily by catalyzing methylation reactions on histones, thereby regulating chromatin structure and gene expression (22). Depending on their substrates, HMTs are categorized into two main types: lysine methyltransferases (KMTs) and protein arginine methyltransferases (PRMTs) (23). KMTs encompassenhancer ofzeste homolog 2 (EZH2), euchromatic histone-lysine N-methyltransferase 2 (EHMT2/G9a), disruptor of telomeric silencing 1-like (DOT1L), SET domain, bifurcated 1 (SETDB1), SET domain, bifurcated 2 (SETDB2), SET domain families, mixed Lineage Leukemia (MLL) families, and others (24). They predominantly catalyze methylation modifications on lysine residues of histone proteins within the context of chromatin regulation (24). PRMTs, comprising PRMT1 to PRMT9, specifically methylate arginines on histones (25). The lysine residues undergo mono-, di-, and trimethylation based on the addition of methyl groups (**Figure 1A**), while arginine residues undergo mono- and dimethylation (26, 27). Different methylation types, catalyzed by various HMTs, result in asymmetric and symmetric dimethylarginines (28, 29). Typical lysine methylation sites are found on histone H3 at lysine 4 (H3K4), 9 (H3K9), 27 (H3K27), 36 (H3K36), and 79 (H3K79), as well as on histone H4 at lysine 20 (H4K20) (30). These modifications regulate a range of chromatin functions (**Figure 1B**). Beyond these classic sites, core histones also possess several less characterized lysine methylation sites (e.g., H3K23me, H3K63me3, H45me1, and H4K12me1).

HMTs are involved in the regulation of gene expression through methylation. For instance, EZH2 catalyzes the trimethylation of H3K27, forming H3K27me3 modifications, thereby suppressing gene transcription (31). In contrast, HMTs like SET domain containing 1A/B (SETD1A/B) are responsible for methylating H3K4, promoting the activation of specific genes (32). This regulation of gene expression plays a crucial role in cellular biology. Beyond directly impacting gene expression, HMTs adjust chromatin structure by altering the methylation of histone tails, affecting chromatin compaction (33, 34). These structural changes subsequently regulate gene accessibility, impacting cellular responses to environmental stimuli and various biological functions, including cell growth and differentiation.

Overall, HMTs participate in multiple cellular biological processes through their complex and finely tuned methylation regulatory network. Their dual mechanism in gene expression regulation and chromatin remodeling provides a precise switch within the complex regulatory networks of the cell. A deeper understanding of these mechanisms is essential for comprehending the fundamental principles of cell biology and the development and progression of tumors, offering a theoretical foundation for the design of new therapeutic strategies.

## 3 Role of HMTs in lung cancer: tumorigenesis and progression

Previous studies have explored the relationship between HMTs and tumorigenesis, indicating a significant regulatory role of HMTs in tumor biology (35). With advancing research, an increasing number of HMTs have been identified as dysregulated in cancer, profoundly influencing tumor phenotypes. Particularly, the expression and aberrant regulation of HMTs are closely associated with the initiation and progression of lung cancer (36). For instance, EZH2, found to be overexpressed in lung cancer tissues compared to normal lung tissues, is significantly associated with the development and progression of lung cancer (37, 38). The level of EZH2 expression correlates positively with the malignancy and poor prognosis in lung cancer (37, 38). Similarly, EHMT2/G9a is overexpressed in aggressive lung cancer cells and is linked to unfavorable prognosis (39). In NSCLC cells resistant to EGFR-TKI, increased expression and enzymatic activity of EHMT2 have been observed (40). Overexpression of G9a, enhancing the focal adhesion kinase (FAK) signaling pathway via the nuclear factor kappa-B (NF-κB) signaling route, promotes invasion and migration in NSCLC cells (41). Other HMTs, such as SET domain containing 8 (SETD8), SET and MYND domain containing 3 (SMYD3), SETDB1, and PRMT5, are also overexpressed in lung cancer, correlating with the tumor’s aggressiveness and clinical characteristics (42–45). Conversely, the downregulation of certain HMTs, such as SET domain containing 8 (SETD2), a tumor suppressor gene significantly reduced in lung cancer, leads to decreased regulation of H3K36me3 modifications, affecting the activity of the signal transducer and activator of transcription 1/interleukin-8 (STAT1/IL-8) signaling pathway. This downregulation promotes epithelial-mesenchymal transition (EMT) in lung adenocarcinoma cells, further facilitating tumor growth and metastasis (46). Additionally, mixed lineage leukemia 4 (MLL4) shows reduced expression in NSCLC tissues and is negatively correlated with disease progression. MLL4 regulates the PI3K/AKT/SRY-related SRY-related HMG-box gene 2 (SOX2) signaling pathway in NSCLC cells, diminishing its role in inhibiting tumor growth and metastasis (47).

The aberrant regulation of HMTs plays a crucial role in lung cancer pathogenesis. For example, EZH2-mediated chromatin remodeling, driven by its specific mark H3K27me3, promotes the transformation of human bronchial epithelial cells (HBEC) into pre-cancerous lesions (48), inducing tumorigenesis. Other studies have shown (49), that SETDB1, as a major histone methyltransferase with oncogenic activity in lung cancer cells, drives lung cancer phenotype by regulating epigenomic landscapes, 3D genome organization, and overall nuclear structure and mechanics. Loss-of-function mutations in SETDB1 can reverse its oncogenic potential (49). In mouse models, MLL deficiency leads to reduced histone H3K4me3, subsequently suppressing the expression of Ras protein-specific guanine nucleotide releasing factor 1 (RASGRF1) and attenuating Kras-driven lung tumorigenesis (50). The Ras oncogene family, activated in most human cancers (51), is a common event in lung adenocarcinoma. In lung adenocarcinoma mouse models, SMYD3 enhances MAP kinase signaling through methylation of MAP3K2, promoting Ras-driven cancer formation (52).

Aberrant regulation of some HMTs is also associated with the metastasis and spread of lung cancer. G9a, for instance, promotes tumor cell growth and invasion in NSCLC by silencing cysteine aspartic acid specific protease (CASP1) and the cell adhesion molecule epithelial cell adhesion molecule (EpCAM) through increased H3K9me2 around promoters (39, 53). SMYD3, by upregulating H3K4me3 in the promoter region of anoctamin-1 (ANO1), promotes ANO1 transcription, thereby facilitating abnormal proliferation of NSCLC cells (54). PRMT5 promotes EMT through the regulation of the EGFR/AKT signaling axis (55). Furthermore, studies have indicated that SETDB1 enhances the migration and invasion capabilities of NSCLC cells by reinforcing the formation of invasive pseudopods and mediating extracellular matrix (ECM) degradation (56).

Tumorigenesis in lung cancer is sometimes accompanied by mutations in methyltransferases. For example, the mutation rate of DOT1L in lung cancer is about 3% (57). The R231Q mutation variant of DOT1L selectively activates the MAPK/ERK signaling pathway in lung cancer cells by enriching H3K79me2 on the RAF1 promoter and epigenetically regulating the expression of downstream targets. This activation subsequently promotes proliferation, colony formation, and migration of lung cancer cells (58). In NSCLC, the mutation rate of MLL2 is 17.5%, and patients with mutant MLL2 have significantly reduced overall survival (59).

In addition to directly modulating histone methylation, certain HMTs may also possess methylation-independent functions, directly influencing the development of lung cancer. For instance, EZH2 has been implicated in augmenting the growth and metastasis of cancer via enhancing the protein levels of mutant p53 variants that drive cancer-driven gain-of-function (GOF) mechanisms (60). EZH2 also exhibits non-catalytic and PRC2-independent roles in stabilizing DNA damage binding protein 2 (DDB2) to facilitate nucleotide excision repair (NER) and control cisplatin resistance in SCLC (61).

Moreover, HMTs can regulate the development of lung cancer through methylation of non-histone substrates. For instance, the transcription factor TWIST1 serves as a non-histone substrate of PRMT1. PRMT1-mediated methylation of twist-related protein 1 (TWIST1) induces EMT, characterized by reduced E-cadherin expression and increased N-cadherin expression, thereby promoting migration and invasion of lung cancer cells (62). In lung cancer cells, downregulation of PRMT5 or overexpression of PRMT1 promotes apoptosis induced by docetaxel and pemetrexed by modulating the degradation of the anti-apoptotic protein CFLARL (63). These findings further underscore the significant role of HMTs in lung cancer.

## 4 Mechanisms of drug resistance mediated by HMTs in lung cancer

**Figure 2:**
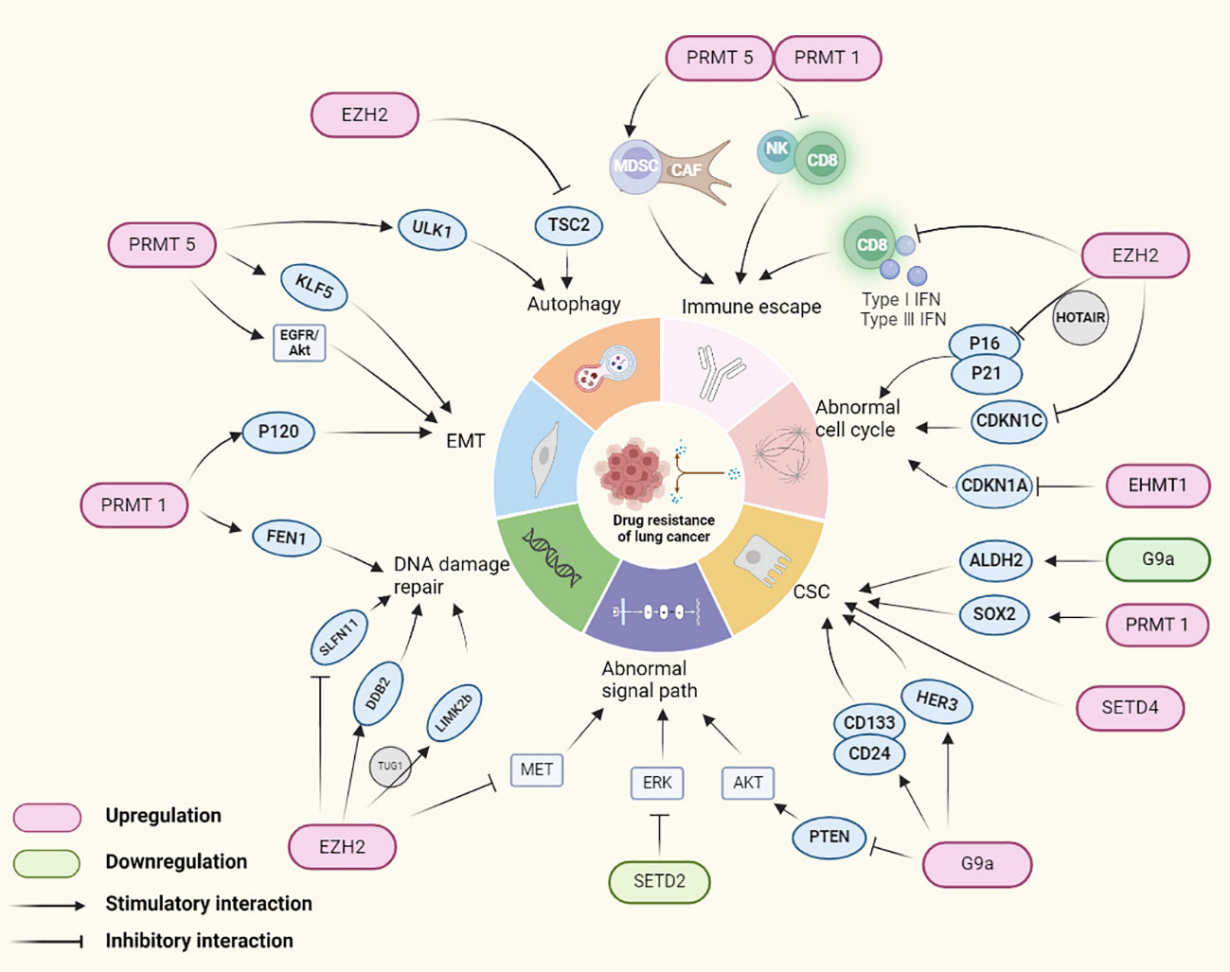
HMTs regulating genes involved in drug resistance mechanisms in lung cancer. In lung cancer, HMTs mediates drug tolerance in lung cancer by altering the expression of lung cancer cell immune escape, cell cycle abnormalities, cancer stem cells, signaling pathways, DNA damage repair, EMT and autophagy related genes.

In the realm of epigenetic histone modifications, HMTs play a pivotal role in gene expression control, and mounting evidence suggests their close association with lung cancer, making them viable targets for drug development. Lung cancer cells often acquire drug-resistant phenotypes through intracellular epigenetic alterations. The role of HMTs in promoting lung cancer drug resistance will be discussed in the following sections, with a summary presented in **Figure 2**.

### 4.1 Immune escape

As one of the effective means against lung cancer, immunotherapy faces the challenge of drug resistance in some patients, characterized initially by effectiveness but gradually diminishing response of the immune system over time, leading to reduced or lost treatment efficacy (7). The immunosuppressive microenvironment plays a key role in the resistance to immune checkpoint inhibitors (ICIs) (64). Studies have shown that controlling interferon-stimulated gene expression is one of the key pathways promoting tumor cell immune resistance (65). EZH2 negatively correlates with type I and III interferons and CD8+ T cell infiltration in NSCLC, altering the tumor microenvironment and inhibiting NSCLC response to programmed death 1 (PD1) blockade (66). High expression of PRMT1 or PRMT5 negatively correlates with immune activation of CD8+ T and NK cells, but positively with the infiltration of myeloid-derived suppressor cells (MDSC) and cancer-associated fibroblasts (CAF), indicating an immunosuppressive microenvironment (67). High expression of PRMT1 and PRMT5 correlates with poorer immunotherapy outcomes in lung adenocarcinoma (LUAD) immunotherapy datasets such as IMvigor210, Kim cohort 2019, and Cho cohort 2020 (67).

### 4.2 Cell cycle abnormalities

Aberrant regulation of the cell cycle can lead to disordered proliferation and development of drug resistance in tumor cells (68). Proteins involved in cell cycle regulation, such as cyclins and kinases, when abnormally expressed, may lead to treatment drug resistance. EZH2, for instance, collaborates with hox transcript antisense intergenic RNA (HOTAIR) to silence the expression of p16 and p21, thereby enhancing NSCLC resistance to gefitinib (69). Furthermore, EZH2 regulates the promoter region of cyclin-dependent kinase inhibitor 1C (CDKN1C) H3K27me3 methylation, promoting its transcriptional silencing and driving NSCLC and SCLC resistance to cisplatin (70, 71). Another study found that the long non-coding RNA urethral epithelium cancer antigen (UCA1) interacts with EZH2, reducing CDKN1A expression, thereby leading to gefitinib resistance (72). Additionally, euchromatic histone lysine methyltransferase 1(EHMT1) downregulation of CDKN1A expression plays a crucial role in lung cancer proliferation (73), indicating the potential for further research on EHMT1 in lung cancer drug resistance. These studies reveal HMTs’ involvement in cell cycle regulation leading to lung cancer drug resistance.

### 4.3 Cancer stem cells

Increasing research suggests that cancer stem cells (CSC) may play a crucial role in promoting cancer drug resistance and metastasis due to their self-renewal and multi-directional differentiation capabilities (74). Aldehyde dehydrogenase (ALDH) is widely used as a marker in CSC (75), and inhibition of EHMT2/G9a promotes transcription of ALDH2, increasing stemness in NSCLC cells and significantly enhancing resistance to paclitaxel (PTX) (76). Overexpression of human epidermal growth factor receptor 3 (HER3) contributes to the formation of CSC-like tumor spheroids. In EGFR-positive lung cancer, upregulation of G9a, through silencing of miR-145-5p, promotes HER3 expression, facilitating EGFR-TKI resistance (77). G9a activity maintains the expression of CD133 and CD24, participating in NSCLC stemness, further promoting tumor-initiating cell (TIC) spheroid formation and growth (78, 79). Inhibition of G9a leads to reduce *in vitro* and *in vivo* stemness and tumorigenicity. In SCLC, increased expression of erythropoietin-producing hepatocellular A2 (EphA2) leads to enhanced expression and activity of PRMT1, bolstering the expression of stemness-related biomarkers SOX2, thereby inducing stemness and chemoresistance in SCLC (80). SETD4 is highly expressed in drug-resistant NSCLC patient cells, regulating CSC in NSCLC patients, contributing to chemoresistance, tumor progression, and poor prognosis (81). These studies reveal that HMTs can influence the expression of CSC marker genes, leading to lung cancer drug resistance.

### 4.4 Abnormal signaling pathways

Under normal conditions, cells regulate proliferation, differentiation, and apoptosis through complex signaling networks (82). However, abnormalities in these signaling pathways can lead to treatment drug resistance in tumor cells (83). Taking NSCLC as an example, the expression of EZH2 shows a negative correlation with MET activation and EGFR-TKI resistance, suggesting that EZH2 may serve as a potential biomarker for EGFR-TKI sensitivity. Studies have revealed that downregulation or inhibition of EZH2 upregulates MET expression and phosphorylation, concurrently enhancing cell proliferation and resistance to EGFR-TKI *in vitro* (84). Additionally, ectopic expression of mutated SETD2 (mtSETD2) induces cisplatin resistance in NSCLC cells by suppressing H3K36me3 and ERK signaling (85). In NSCLC, upregulation of EHMT2 leads to downregulation of phosphatase and tensin homolog (PTEN), promoting activation of the AKT pathway and consequently facilitating resistance to EGFR-TKI (40).

### 4.5 DNA damage repair

DNA damage repair mechanisms play a crucial role in maintaining genomic stability in normal cells. However, hyperactive repair systems in tumor cells might be a fundamental cause of drug resistance (86). For instance, EZH2 facilitates platinum resistance in SCLC by stabilizing DDB2 and promoting NER (61). Flap endonuclease 1 (FEN1), playing a key role in DNA replication and repair (87), is maintained at high expression levels by PRMT1, crucial for DNA repair and chemotherapy resistance in lung cancer cells (88). Studies indicate that the expression of Schlafen11 (SLFN11) is closely related to the sensitivity to DNA-damaging agents (89). Under chemotherapy induction, EZH2-mediated H3K27me3 deposition in the SLFN11 gene body suppresses SLFN11 expression, thereby enhancing DNA repair efficiency, enabling tumor cell adaptation and survival (90). Additionally, different phases of the cell cycle impact DNA damage repair. G1 and G2 phases are critical for DNA damage repair, and repairs during these phases may lead to chemotherapy resistance in tumor cells. For example (91), the splicing variant of LIM kinase 2 (LIMK2b), a direct target of p53, involved in cell proliferation and division control, plays a crucial role in promoting the G2/M DNA damage checkpoint. EZH2 upregulates LIMK2b expression, promoting growth and chemotherapy resistance in SCLC resistant cells (platinum, doxorubicin, etoposide) (92). These findings highlight the profound connection between HMTs-involved DNA damage repair and tumor drug resistance.

### 4.6 EMT

EMT participates in the development of tumor drug resistance, providing tumor cells with a more treatment-tolerant state by affecting cell characteristics, microenvironment, and survival mechanisms (93). PRMT1 mediates EMT by methylating Twist-1 and increasing p120-catenin expression, enhancing the invasiveness of NSCLC and consequently promoting osimertinib resistance (94). On the other hand, PRMT5, as a key oncogenic regulator, promotes the EMT process in human lung cancer cells through the EGFR/AKT signaling axis, thereby exacerbating resistance development (95).

### 4.7 Autophagy

In cancer therapy, therapeutic drugs typically eliminate tumor cells by inducing cell death. However, tumor cells might escape therapy-induced cell death by initiating autophagy (96). For instance, EZH2-mediated H3K27me3 trimethylation suppresses the expression of tuberous sclerosis complex 2 (TSC2), thereby inhibiting autophagy and reducing NSCLC cell resistance to cisplatin (97). Unc-51 like autophagy activating kinase 1 (ULK1), a key regulator of autophagy in lung cancer cells, is methylated by overexpressed PRMT5, enhancing the autophagic process and improving the survival rate of lung cancer cells under hypoxic conditions (98), promoting resistance to carboplatin.

### 4.8 Others

Neuroendocrine differentiation (NED), a key process in the transformation of cancer cells into neuroendocrine-like cells post-treatment, is widely considered one of the significant mechanisms of acquired treatment resistance (99). Studies show that in NSCLC, PRMT5 plays a key role in chemotherapy-induced NED, and targeting PRMT5 effectively restores sensitivity to etoposide in NSCLC cells (100). On the other hand, SMYD2 is overexpressed in drug-resistant LUAD cell lines, mediating cisplatin resistance through epigenetic regulation of p53 (101). Additionally, some HMTs also influence chemotherapy resistance. For example, the DOT1L R231Q mutation significantly reduces cell sensitivity to cisplatin, vincristine, and the small molecule inhibitor SGC0946 (58), though its exact mechanisms remain to be explored, providing a useful direction for further research.

## 5 HMTs inhibitors in lung cancer treatment

HMT inhibitors are emerging as a novel approach in cancer therapy (102), leading to the development of numerous inhibitors. Most work by competitively binding to the S-adenosylmethionine (SAM) binding sites, thereby inhibiting HMT activity and affecting histone methylation levels (103). This regulation can impact gene expression and cell function, playing a crucial role in tumor development and progression.

Recent studies have found that HMT inhibitors show significant potential in treating NSCLC and SCLC. For example, the EZH2 inhibitor DZNep inhibits NSCLC cell proliferation, induces apoptosis, and causes G1 phase arrest (104). Other EZH2 inhibitors like GSK343 (105) and PRMT5 inhibitors [GSK591 (106), PF-06939999 (107)] also demonstrate inhibitory effects on A549 cells. Novel EZH2 inhibitors such as 6Y reduce EZH2 expression and induce cell cycle arrest at G2/M phase (108). In SCLC, EPZ-6438 inhibits proliferation and diminishes the chronic inflammatory impact of the senescence-associated secretory phenotype (SASP) on the cancer microenvironment (109). Additionally, HMT inhibitors play a key role in promoting apoptosis. For instance, EZH2 inhibitors GSK343 or DZNep induce apoptosis in A549 and H1299 cells by downregulating the phosphorylation of EGFR and AKT (110).

In targeting specific mutant forms of lung cancer, HMTs inhibitors can also exhibit a certain level of efficacy. At the transcriptional and epigenetic regulation levels, EZH2 inhibitors DZNep and EPZ6438 reduce the transformation of human bronchial epithelial cells (HBEC) into pre-cancerous lesions and achieve transcriptional reprogramming (111). At the transcriptional and epigenetic regulation levels, EZH2 inhibitors DZNep and EPZ6438 reduce the transformation of HBEC into pre-cancerous lesions and achieve transcriptional reprogramming (48).

Moreover, the EZH2 inhibitor DZNep shows significant therapeutic potential in mouse models, notably slowing the growth rate of lung adenomas in A/J mice by 55% (48). Other inhibitors like G9a inhibitors UNC0642 (78) and UNC0638 (21), and the specific PRMT5 inhibitor GSK591 (95), have demonstrated growth-inhibitory effects on lung adenocarcinoma in xenograft mouse models.

In lung cancer therapy, apart from the significant effects of individual HMT inhibitors, various combination regimens have shown notable synergistic effects both *in vitro* and *in vivo*, including with chemotherapy and immunotherapy agents. For instance, the specific PRMT5 inhibitor GSK591 combined with anti-PD-L1 therapy shows significantly enhanced efficacy in treating LLC mouse models compared to anti-PD-L1 therapy alone (112).

Despite these HMT inhibitors showing anti-tumor activity in *in vitro* and *in vivo* experiments against lung cancer, clinical trials specifically targeting HMT inhibitors in lung cancer therapy remain limited. For example, the clinical trial of the EZH2 inhibitor EPZ-6438 (Tazemetostat) combined with topotecan and pembrolizumab for treating recurrent SCLC has entered Phase I (Trial ID: NCT05353439). Similarly, clinical trials of the oral PRMT1 inhibitor CC-90011 combined with platinum-based chemotherapy in SCLC are ongoing (Trial ID: NCT03850067). Additionally, PRMT1 inhibitor GSK3368715 and EZH2 inhibitor GSK2816126 have achieved satisfactory positive results in solid tumors (Trial IDs: NCT03666988, NCT02082977), demonstrating their potential research value in lung cancer.

## 6 Impact of HMT inhibitors on lung cancer drug resistance

HMT inhibitors serve not only as direct therapeutic targets for lung cancer but also influence lung cancer drug resistance. These inhibitors may reverse cancer cells’ resistance to traditional chemotherapy and targeted therapies (**Table 1**). For example, in SCLC, the EZH2 inhibitor GSK126 reduces platinum and etoposide resistance induced by chromodomain Y-like (CDYL) through inhibiting CDKN1C (70). Studies suggest (115) that in EGFR wild-type NSCLC patients, conventional EGFR-TKI treatments are ineffective, but using EZH2 inhibitors (such as GSK343 or DZNep) sensitizes lung adenocarcinoma cells to gefitinib (110). Additionally, the inhibitor EPZ-6438 (116) inactivates EZH2, increasing A549 cell’s chemosensitivity to cisplatin. Similarly, the PRMT5 inhibitor C9 (98) significantly enhances lung cancer cells’ sensitivity to carboplatin. These findings indicate the potential role of HMT inhibitors in reversing drug resistance in lung cancer treatment by modulating gene expression and increasing tumor cell sensitivity to therapy.

**Table 1:** The impact of HMT inhibitors on drug resistance in lung cancer.

HMTs	Inhibitor	Experimental object	Types of lung cancer	Role of drug resistance in lung cancer(Reference)
EZH2	GSK126	H69 cells	SCLC	Reduced resistance to cisplatin and etoposide (70)
	GSK343 DZNep	Wild-type EGFR cells (A549 and H1299)	LUAD	Increased sensitivity to gefitinib (110)
	GSK343	Gefitinib-resistant PC9 cells	NSCLC	Inhibits cell viability, proliferation, and promotes apoptosis (113)
	EPZ011989	chemotherapy-resistant xenograft mice	SCLC	Restores sensitivity to irinotecan (90)
G9a	UNC0642 UNC0638	Erlotinib-resistant PC9 xenograft SCID mice	NSCLC	Restores sensitivity to erlotinib (40)
DOT1L	SGC0946	H1299 xenograft mice with DOT1L R231Q mutation	NSCLC	Reduces resistance to cisplatin and vinorelbine (58)
SMYD3	EPZ031686	DMS-114 cells	SCLC	Restores sensitivity of SCLC to alkylating chemotherapy (114)
SMYD2	BAY-598	Cisplatin-resistant H460 cells	SCLC	Restores sensitivity to cisplatin (101)

Combining HMT inhibitors with other therapeutic methods (such as chemotherapy, immunotherapy, etc.) may create more effective treatment regimens, especially in addressing drug resistance. For instance, the combination of the EZH2 inhibitor GSK343 with gefitinib inhibits cell viability, proliferation, and promotes apoptosis in gefitinib-resistant PC9 cells (113). In SCLC-resistant xenograft mouse models, the EZH2 inhibitor EPZ011989 restores tumor sensitivity to irinotecan by upregulating SLFN11, and its combination with irinotecan significantly inhibits tumor growth (90). Similarly, G9a inhibitors UNC0642 and UNC0638 sensitize resistant NSCLC cells *in vivo* to TKIs by inhibiting the AKT pathway, and their combination with erlotinib markedly suppresses tumor growth in transplanted mice (40). Specific DOT1L inhibitors like EPZ5676, EPZ004777, and SGC0946, as well as over 20 other small molecule inhibitors, have shown significant inhibitory effects on NSCLC cells with the DOT1L R231Q mutation when combined with chemotherapy drugs (117). The R231Q mutation of DOT1L induces resistance of lung cancer cells to cisplatin and vinorelbine both *in vitro* and *in vivo*. The DOT1L small-molecule inhibitor SGC0946 reverses drug resistance *in vivo*, enhancing its anti-tumor activity (58). The newly synthesized inhibitor CM-1 demonstrates the ability to enhance sensitivity of DOT1L R231Q mutant NSCLC cells to EGFR-TKIs and chemotherapy, potentially reversing resistance to these treatments (117). Additionally, the SMYD3 inhibitor EPZ031686, by reducing the methylation level of ring finger protein 113A (RNF113A), makes SCLC cells more sensitive to alkylating agents and promotes sustained response to chemotherapy (114). The SMYD2 inhibitor BAY-598 not only enhances the sensitivity of cisplatin-resistant NCI-H460 cells to cisplatin but also inhibits cell migration and tumor sphere formation (101). HMT inhibitors may enhance the toxicity of treatments to cancer cells, potentially overcoming these resistances and inhibiting the malignant phenotype of lung cancer cells.

Studies on the role of HMT inhibitors in resistance have entered clinical trial phases, with the EZH2 inhibitor EPZ-6438 (Tazemetostat) undergoing trials in platinum-resistant small cell lung cancer patients (Trial ID: NCT05353439). As an EZH2 inhibitor, EPZ-6438 holds the potential to reverse cancer cell resistance to platinum-based chemotherapy drugs by modulating histone methylation levels and affecting related gene expression.

## 7 Summary and prospect

HMTs play a crucial role in gene regulation, and their aberrant activity can lead to malignant transformation in lung cancer cells (36).Studies have shown that certain HMTs are overexpressed in lung cancer patients (39), potentially leading to abnormal gene methylation and thus regulating lung cancer cell growth, differentiation, and drug resistance (48). By comprehensively examining the expression patterns of HMTs in different subtypes and grades of lung cancer patients, we can better understand their relationship with cancer development and treatment response.

Inhibiting or modulating HMT activity could emerge as a promising strategy for lung cancer treatment, potentially slowing cancer progression or enhancing patient sensitivity to treatment. While many HMT inhibitors have already shown effectiveness in various cancers (102) and some even help reverse tumor cell drug resistance (118), only a few have been confirmed effective in lung cancer drug resistance. There are several major obstacles to overcome: Firstly, lung cancer is a heterogeneous tumor with diverse molecular mechanisms, including various subtypes and molecular subgroups. Thus, HMT response may vary depending on the lung cancer subtype, rendering inhibitors effective in some situations but not in others. The patient’s treatment history, including prior radiotherapy, chemotherapy, or targeted therapy, could also affect HMT expression. Secondly, lung cancer’s drug resistance mechanisms are highly diverse, including cell cycle regulation, DNA damage repair, apoptosis, and more. Therefore, a single HMT inhibitor might not adequately address the variety of resistance mechanisms. Lastly, some HMT inhibitors may not have undergone sufficient clinical research and validation, necessitating a deeper understanding of their mechanisms, specificity, and effects in different subtypes and clinical backgrounds to ensure their safety and efficacy.

To address these challenges, more in-depth research and clinical trials are needed. Specifically, the development of more specific HMT inhibitors targeting various molecular subtypes of lung cancer could enhance the precision of treatment. Using combination therapy strategies, such as combining HMT inhibitors with other treatments (e.g., chemotherapy, immunotherapy, or targeted therapy), might produce synergistic effects, enhancing the overall effectiveness of treatment and potentially overcoming drug resistance. However, optimization of combination therapy strategies, including adjustments in drug dosages and sequences, is necessary to maximize their potential in reversing resistance. Given lung cancer’s heterogeneity and various mutations or genetic alterations, including epigenetic changes, developing compounds targeting multiple targets is essential. Ultimately, personalized treatment plans based on the patient’s molecular characteristics and resistance mechanisms could better address the complexity and heterogeneity of lung cancer. Overall, treating drug resistance remains a major challenge in the field of lung cancer treatment, and further investigation into epigenetic drugs, particularly HMT inhibitors, may offer new therapeutic avenues to overcome this challenge.

## Author contributions

LZ: Writing – original draft. XZ: Writing – original draft. YS: Writing – original draft. YN: Writing – original draft. JF: Writing – original draft. ZJ: Writing – original draft. WL: Writing – original draft. XW: Writing – review & editing. NW: Writing – review & editing.
